# Two different mechanisms mediate chemotaxis to inorganic phosphate in *Pseudomonas aeruginosa*

**DOI:** 10.1038/srep28967

**Published:** 2016-06-29

**Authors:** Miriam Rico-Jiménez, Jose Antonio Reyes-Darias, Álvaro Ortega, Ana Isabel Díez Peña, Bertrand Morel, Tino Krell

**Affiliations:** 1 Department of Environmental Protection, Estación Experimental del Zaidín, Consejo Superior de Investigaciones Científicas, Granada, Spain; 2 Department of Physical Chemistry, Faculty of Chemistry, Regional Campus of International Excellence “Campus Mare Nostrum”, University of Murcia, 30071 Murcia, Spain

## Abstract

Inorganic phosphate (Pi) is a central signaling molecule that modulates virulence in various pathogens. In *Pseudomonas aeruginosa*, low Pi concentrations induce transcriptional alterations that increase virulence. Also, under low Pi levels, *P. aeruginosa* exhibits Pi chemotaxis—a process mediated by the two non-paralogous receptors CtpH and CtpL. Here we show that the two receptors operate via different mechanisms. We demonstrate that the ligand binding domain (LBD) of CtpH but not CtpL binds Pi directly. We identify the periplasmic ligand binding protein PstS as the protein that binds in its Pi loaded state to CtpL, resulting in receptor stimulation. PstS forms part of the Pi transporter and has thus a double function in Pi transport and chemotaxis. The affinity of Pi for CtpH was modest whereas that for PstS very high, which may explain why CtpH and CtpL mediate chemotaxis to high and low Pi concentrations, respectively. The *pstS/ctpH* double mutant was almost devoid of Pi taxis, indicating that PstS is the only CtpL Pi-shuttle. Chemotaxis mechanisms based on indirect ligand recognition were unambiguously identified in enterobacteria. The discovery of a similar mechanism in a different bacterial order, involving a different chemoreceptor type and chemoeffector suggests that such systems are widespread.

*P. aeruginosa* is a ubiquitously occurring microorganism that is capable of causing human opportunistic infections^1^. As such, *P. aeruginosa* is the leading cause of nosocomial infections, particularly in immunocompromised, cancer, burn and cystic fibrosis patients^2^. *P. aeruginosa* chronic lung infections are the major cause of death in cystic fibrosis patients^3^. Furthermore, *P. aeruginosa* is the number one pathogen involved in ventilator associated pneumonia and burn wound infections—both of which are associated with very high mortality rates^4^. This, combined with the emergence of strains resistant to all commercially available antibiotics, makes *P. aeruginosa* one of the most feared pathogens^5^.

Pi was identified as a central virulence signal in *P. aeruginosa*: Pi starvation was found to elicit important transcriptional changes in genes related to Pi acquisition, quorum sensing, chemotaxis, toxin secretion and regulation, which led to increased virulence-associated phenotypes, including motility and cytotoxicity^6^. Pi depletion was found to cause *P. aeruginosa* to shift towards expression of a lethal phenotype against *C. elegans*^7^. Following surgical injury, Pi becomes rapidly depleted within intestinal mucus to levels that are associated with the expression of important virulence determinants in *P. aeruginosa* and it was proposed that post-operative Pi administration may be a convenient strategy to contain pathogens^8^. As a consequence, severe hypophosphatemia in sepsis has become a predictor of mortality^9^. This Pi mediated regulation of virulence has also been observed for other pathogens, for example, *Agrobacterium tumefaciens*^10^, *Vibrio cholerae*^11^ and *Bacillus anthracis*^12^.

Findings from the Kato laboratory have revealed that *P. aeruginosa* exhibits chemotaxis to Pi^13^,^14^,^15^. Interestingly, taxis is mediated by two chemoreceptors, named CtpL and CtpH, that respond to low and high Pi concentrations, respectively^14^. Chemotaxis was only observed at low Pi concentrations^14^ and the transition from high to low Pi concentrations caused an increase in the expression of both receptors and of proteins that participate in chemotactic signaling^6^, indicating that Pi chemotaxis may be related to virulence. Chemoreceptors are at the beginning of chemosensory signaling cascades and integrate the chemoeffector signal either by direct recognition or by binding to chemoeffector-engaged periplasmic proteins^16^.

Interestingly, the LBDs of CtpL and CtpH are unannotated in InterPro, differ in size and secondary structure prediction and share no significant sequence similarities (Figs S1–S3), indicating that there are two non-paralogous chemoreceptors that mediate chemotaxis to the same chemoeffector. This fact, along with the clinical relevance of Pi, has motivated the current study into molecular mechanisms of CtpL and CtpH mediated Pi taxis. Here, we show that the receptors operate via two different mechanisms and that CtpL mediated taxis is linked to Pi uptake.

## Results

### CtpH binds Pi, while CtpL does not bind Pi

**Figure 1:**
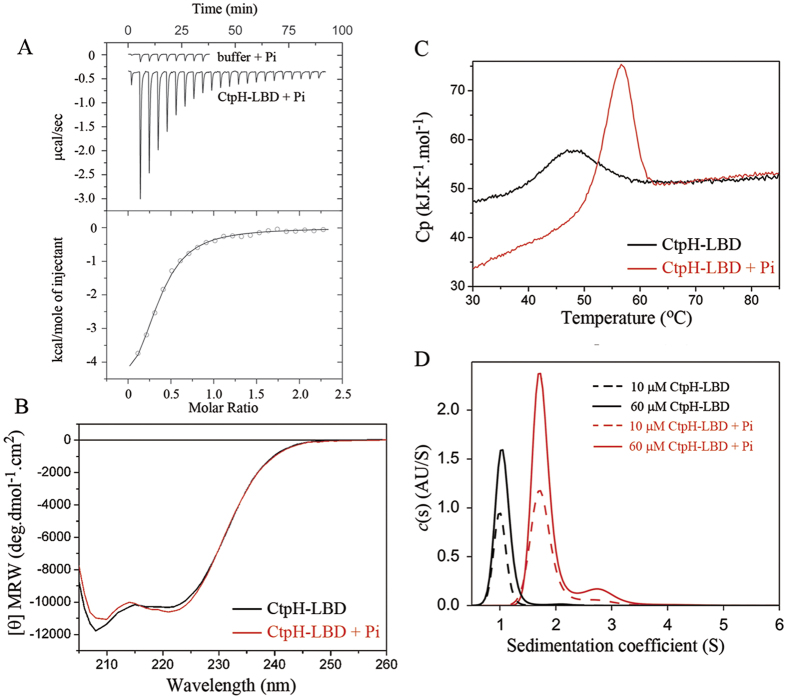
The interaction of CtpH-LBD with Pi. (**A**) Microcalorimetric titration of buffer or 172 μM CtpH-LBD with 3 mM Pi. Lower panel: corrected, integrated titration data and curve fit. (**B**) CD spectra of CtpH-LBD (51 μM) in the absence and presence of 5 mM Pi. (**C**) DSC study of CtpH-LBD (51 μM) in the absence and presence of 5 mM Pi. (**D**) Sedimentation velocity analyses (45 000 rpm) of CtpH-LBD in the absence and presence of 500 μM Pi.

To establish whether CtpH and CtpL recognize Pi directly, the LBDs of both receptors (Fig. S1) were expressed and purified from the soluble fraction of *Escherichia coli* lysates. Purified proteins were then submitted to Isothermal Titration Calorimetry (ITC) binding studies. As shown in Fig. 1A, the injection of 3 mM Pi into dialysis buffer gave rise to small and uniform peaks that are due to dilution effects. However, the titration of CtpH-LBD with Pi produced large heat changes that diminished as protein saturation advanced.

Data analysis revealed that the binding of CtpH-LBD with Pi has a *K*_D_ of 22 μM (Table 1). In contrast, the titration of CtpL-LBD with Pi, conducted at different temperatures, revealed no binding. We hypothesized that the ligand binding site at CtpL-LBD may be occupied with tightly bound Pi. To verify this hypothesis, CtpL-LBD was denatured, dialyzed and refolded. However, ITC analyses of the resulting protein did not show any Pi binding, suggesting that CtpL does not recognize Pi directly.

### CtpH-LBD binds to other compounds that have a pyrophosphate moiety

To determine the ligand specificity of CtpH-LBD, we conducted ITC studies with a number of phosphorylated compounds. We found that CtpH-LBD binds to pyrophosphate with an affinity only slightly less than its affinity for Pi (Table 1). This is consistent with a study from Kato *et al*.^13^, which demonstrated *P. aeruginosa* chemotaxis towards millimolar concentrations of pyrophosphate. We also found that CtpH-LBD binds to other compounds with a pyrophosphate moiety, including ATP and ADP (Table 1). In contrast, binding experiments with compounds containing a single phosphate group (Table 1) showed no binding. Because it has been previously shown that Pi starved cells are attracted to arsenate and that Pi was shown to inhibit taxis towards arsenate^17^, we also studied arsenate and arsenite binding. However, when titrated with 5 mM arsenate or arsenite, CtpH-LBD did not bind to either compound. In summary, our data indicates that CtpH-LBD binds at physiologically relevant affinities to Pi and to other compounds that have a pyrophosphate group.

### Pi induces dimerization and stabilization of CtpH-LBD

To shed light on CtpH sensing mechanisms, CtpH-LBD was subjected to different biophysical techniques. The far UV circular dichroism (CD) spectrum for CtpH-LBD (Fig. 1B) showed minima at 208 and 222 nm, typical of α-helical proteins. No major spectral changes were observed when Pi was added, indicating that ligand binding does not significantly alter CtpH-LBD secondary structure.

Thermal unfolding of the protein was monitored by Differential Scanning Calorimetry (DSC). In the absence of Pi, protein unfolding was characterized by a single event with an enthalpy change (Δ*H*) of 28.2 + /−0.4 kcal/mol and a Tm of 46.9 + /−0.2 °C (Fig. 1C). The saturation of the protein with Pi caused a large Tm increase (Tm = 56.6 + /−0.2 °C) accompanied by an increase in Δ*H* to 39.9 + /−1.5 kcal/mol. As shown in Fig. S4, protein unfolding was highly reversible.

The consequences of Pi binding on the oligomeric state of CtpH-LBD were determined by analytical ultracentrifugation (AUC). A sedimentation velocity analysis was carried out for CtpH-LBD in the presence and absence of Pi and the resulting sedimentation profiles are shown in Fig. 1D. In the absence of Pi, a single peak with an S value (standardized to 20 °C in water) of 1.95 was observed, which corresponds to a protein monomer and molar mass of 21.4 kDa. In the presence of Pi, no peak corresponding to the monomer was observed; furthermore, the sedimentation profile showed one major peak (86% of the sedimentation material) at an S of 3.25 (corresponding to a molecular weight of 37.1 kDa) and a second minor peak at an S of 5.16 (corresponding to a molecular weight of 78.4 kDa). Thus, in the presence of Pi, both of the identified molecular weights are very close to the sequence-derived masses of the dimer (39,566 Da) and tetramer (79,132 Da). To confirm these results, sedimentation equilibrium experiments were carried out in the presence and absence of Pi (Fig. S5). Analysis of the gradient profiles revealed average molecular weights of 20,000 ± 300 Da in the absence of Pi, and 39,100 ± 300 Da in its presence. Taken together, this data reveals that Pi binding causes CtpH-LBD dimerization, accompanied by a large stability increase.

### Pi signal input to CtpL occurs via PstS

**Figure 2:**
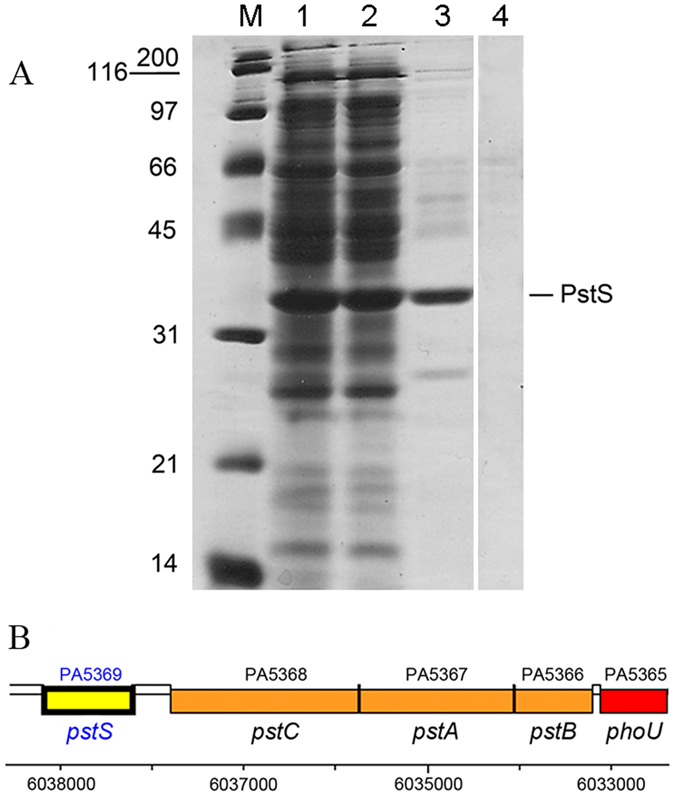
Identification of PstS as a CtpL-LBD ligand. (**A**) SDS-PAGE gel of a pull-down experiment on immobilized CtpL-LBD. Lane 1: Protein extract of *P. aeruginosa* applied to the column. Lane 2: Flow through of this column. Lane 3: Proteins eluted. Lane 4: Protein eluted in control experiment (i.e. the same protocol except that no protein was immobilized on the column) (**B**) Genetic organization of the *pstSCAB* genes encoding the Pi transporter.

Based on the absence of Pi binding to CtpL-LBD, we hypothesized that signal input to CtpL occurs via a Pi-loaded protein. To verify this hypothesis, we conducted pull-down assays in which CtpL-LBD was immobilized on a column as bait. Subsequently, a protein extract of *P. aeruginosa,* grown under Pi limiting conditions, was applied to the column (Fig. 2A, lane 1) and the flow-through was collected (Fig. 2A, lane 2).

After column washing, a buffer containing 6 M guanidine hydrochloride was applied to the column. This chaotropic agent causes complete protein unfolding and the release of any bound prey. SDS-PAGE gel electrophoresis of the eluate showed one major band with an approximate weight of 35 kDa (Fig. 2A, lane 3). When the same procedure was repeated without immobilized bait, this protein was not present (Fig. 2A, lane 4, Fig. S6).

The eluted protein was identified as PstS (PA5369) by mass spectrometry based peptide mapping and matching with theoretical protein digests. This identification was unambiguous as evidenced by a MASCOT score of 759 (scores above 88 are significant) and an excellent sequence coverage of 88%. In addition, the sequence derived molecular weight of PstS (34,474 Da) coincided with that determined by SDS-PAGE. Figure 2A also shows that PstS is the most abundant protein in *P. aeruginosa* when grown under Pi limiting conditions—an observation that has been made previously^18^.

The *pstS* gene encodes a periplasmic Pi-binding protein and is adjacent to the *pstABC* genes encoding the Pi transporter (Fig. 2B)^19^. The molecular mechanism of this transporter has been elucidated in *E. coli*. PstA and PstC are integral membrane proteins and PstB is bound at the cytosolic side to the PstAC complex. The periplasmic PstS protein binds to Pi and, when bound to the PstAC complex, delivers Pi to be transported^20^.

### Pi binds with very high affinity to PstS

To study ligand recognition, PstS was overexpressed in *E. coli* and purified. However, ITC biding studies using Pi and other phosphorylated compounds showed no binding. We hypothesized that PstS may have been co-purified with a tightly bound ligand. To explore this possibility, we unfolded, dialyzed and refolded PstS. Analysis of the refolded protein by far UV CD spectroscopy indicates that it has a very similar secondary structure content as compared to the protein obtained after affinity purification (Fig. S7, Table S1). Intrinsic tryptophan fluorescence spectroscopy also showed that the emission spectra of the refolded protein and that obtained after purification are very similar, indicative of a very similar protein folding (Fig. S8). In addition, dynamic light scattering measurements of both protein samples showed almost identical hydrodynamic radii and only minor amounts of aggregates (Fig. S9).

**Figure 3:**
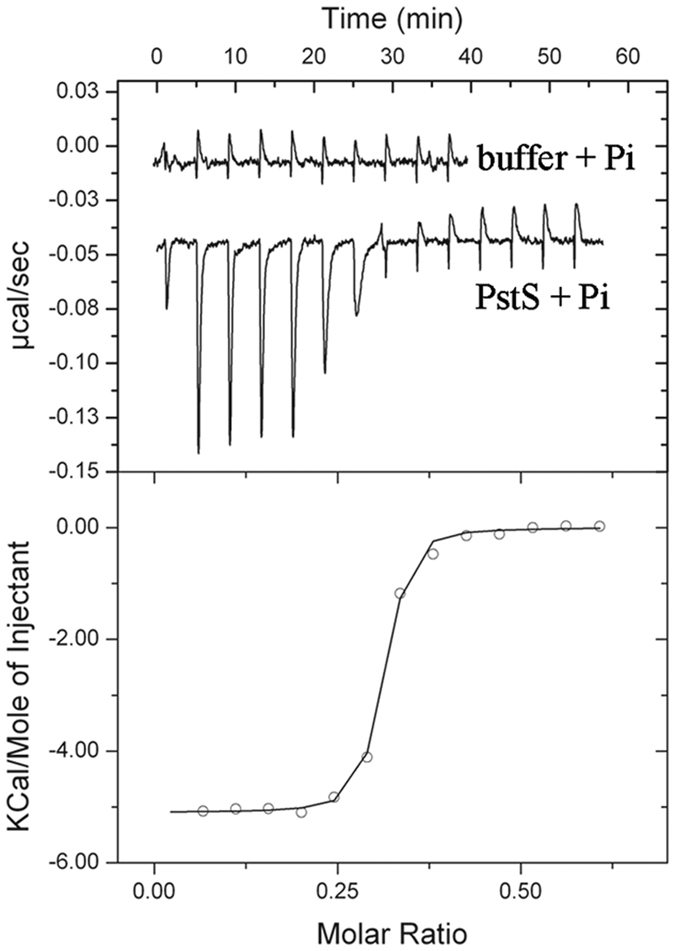
ITC analysis of the binding of PstS and Pi. Upper panel: microcalorimetric titration of buffer or 10 μM PstS with 200 μM Pi. Lower panel: corrected, integrated titration data and curve fit.

ITC analysis on the resulting protein revealed that PstS binds Pi with an ultra-high affinity (*K*_D_ = 7 ± 1 nM; Fig. 3). Binding was driven by favorable enthalpy (Δ*H* = −5.1 ± 0.1 kcal/mol) and entropy changes (*T*Δ*S* = 6.0 ± 0.1 kcal/mol), which is a binding mode frequently seen for ultra-tight interactions^21^. The n-value of 0.29 ± 0.02 indicates that only a part of the protein is able to bind Pi. The above mentioned structural similarity between the refolded protein and that obtained after purification suggests that the protein fraction that is unable to bind Pi corresponds to correctly folded protein. Most likely, during refolding this protein has acquired Pi, which is present as an impurity in the reagents of the refolding buffer. We found that PstS also binds to pyrophosphate and ATP, although with much lower affinities (Table 1). Similarly to CtpH-LBD, compounds harboring a single phosphoryl group (legend of Table 1) failed to bind.

### PstS forms a 1:1 complex with CtpL-LBD

**Figure 4:**
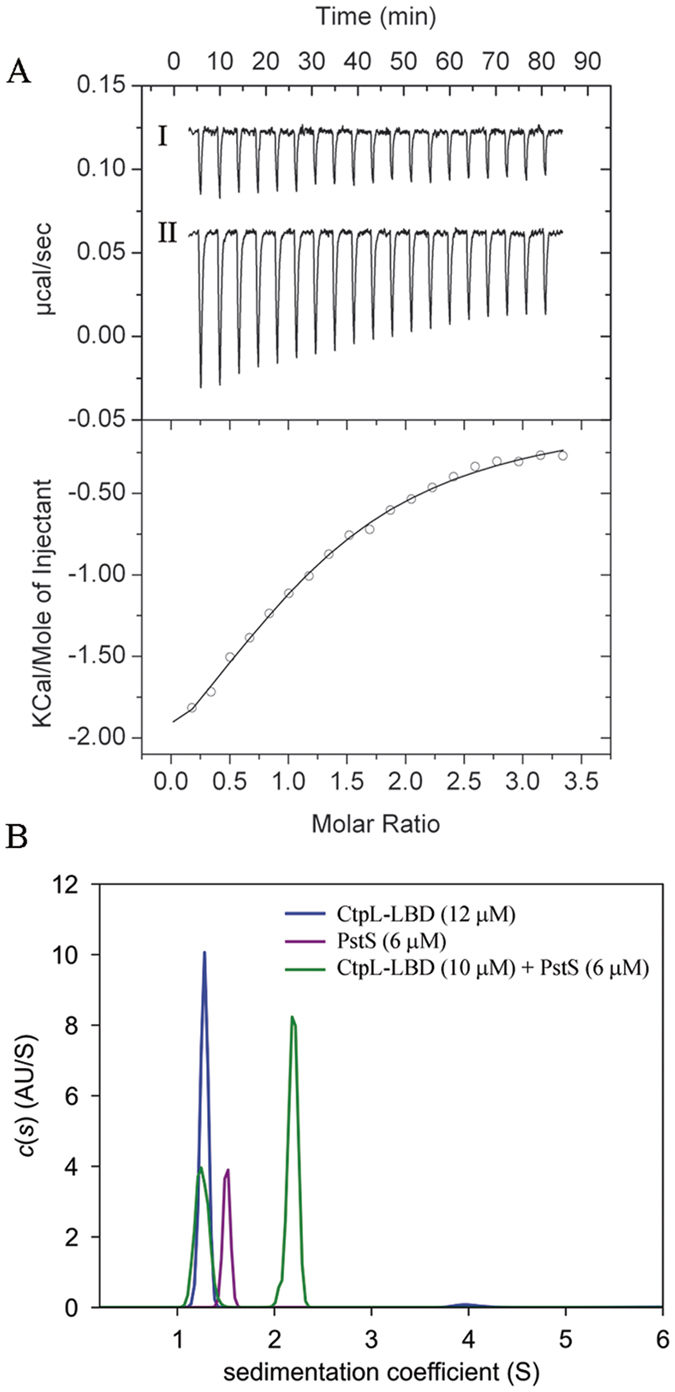
Characterization of the interaction between PstS and CtpL-LBD. (**A**) Microcalorimetric titration of buffer (I) or 5 μM PstS (II) with 80 μM CtpL-LBD. Lower panel: corrected, integrated peak areas of titration data and fit. (**B**) Sedimentation velocity study of individual PstS and CtpL-LBD and a mixture of both proteins.

To characterize signal recognition at CtpL, we conducted microcalorimetric titrations of Pi-loaded PstS with CtpL-LBD (Fig. 4A). Whereas the buffer control produced small peaks of comparable size, a hyperbolic binding curve was obtained for the titration of PstS with CtpL-LBD. Data analysis revealed a *K*_D_ of 3.7 ± 0.3 μM.

Sedimentation velocity analyses were carried out for CtpL-LBD and PstS individually and as a mixture (Fig. 4B). S values of 2.2 and 2.5 were observed for CtpL-LBD and PstS, respectively. The respective molar masses of 31 kDa and 33 kDa are very close to the sequence derived masses of monomeric forms of CtpL-LBD (33,097 Da) and PstS (36,636 Da), respectively. When CtpL and PstS were mixed together, the mixture contained two different sedimenting species. A slower species with an S value of 2.2 corresponded to excess CtpL-LBD, and a species with an S value of 3.7, representing the CtpL-LBD/PstS complex. Data analysis revealed that this second species corresponds to a 1:1 complex between CtpL-LBD and PstS.

### Deletion of the *pstS* gene strongly reduces chemotaxis to low Pi concentrations

To assess the role of PstS in chemotaxis, we conducted quantitative capillary chemotaxis assays toward low (0.01 and 0.1 mM) and high (10 mM) Pi concentrations, which corresponded to the Pi concentrations used in the initial characterization of both receptors^14^. To this end, we created a *pstS* deletion mutant in the wild type (wt) and *ctpH* mutant backgrounds. For complementation studies, *pstS* was cloned into plasmid pBBR1MCS-5, which was then introduced into the *pstS* mutant. Further strains included in this analysis were wt, the wt complemented with plasmid pBBR1MCS-5 and the *ctpH* mutant.

**Figure 5:**
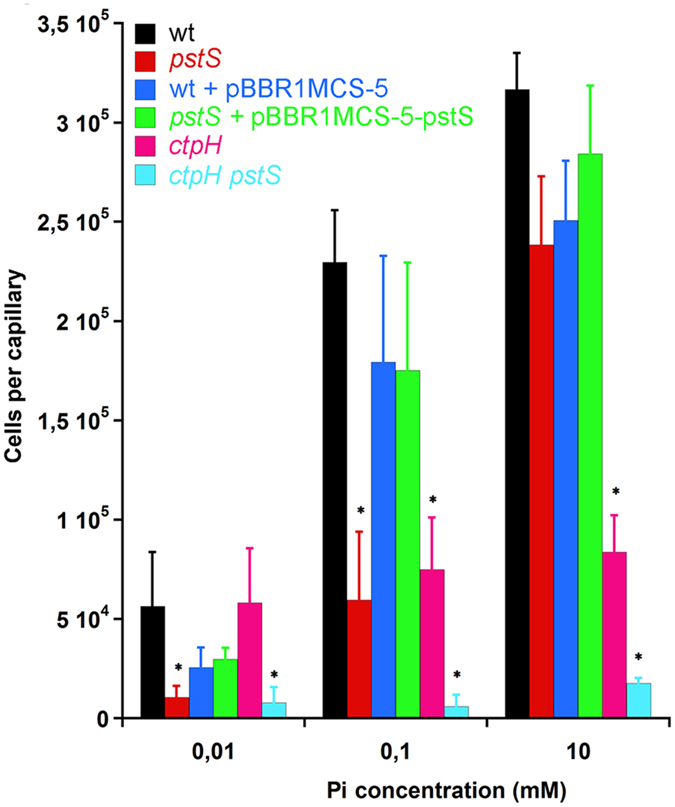
Capillary chemotaxis assays of different *P. aeruginosa* PAO1 strains towards different concentrations of Pi. Means and standard deviations from three biological replicates conducted in triplicate. Data were corrected with the number of bacteria that swam into buffer-containing capillaries (810 ± 149). *P < 0.05, Student’s t-test of *pstS*, *ctpH* and *ctpH/pstS* mutant with respect to the wt strain. No statistically relevant differences were observed between the two plasmid containing strains.

As shown in Fig. 5, deletion of *pstS* dramatically reduced chemotaxis to low Pi concentrations. The chemotactic response was restored upon complementation with *pstS* and was similar to that of the wt strain with empty plasmid pBBR1MCS-5. Mutation of *pstS* did not greatly affect chemotaxis to high Pi concentrations. Mutation of *ctpH* compromised chemotaxis to 0.1 and 10 mM Pi, whereas no significant changes were observed to 0.01 mM Pi—a finding that agrees with previously published data^14^. Finally, only very marginal Pi chemotaxis was observed in the *pstS*/*ctpH* double mutant, which also agrees with previous work that reported the absence of Pi taxis in a *ctpL/ctpH* mutant^14^.

## Discussion

When *P. aeruginosa* growing in high concentrations of Pi is exposed to low Pi concentrations, significant transcriptional level alterations occur that lead to induction of virulence determinants^6^,^7^,^8^. Because *P. aeruginosa* was found to exclusively show chemotaxis to Pi when grown under low Pi concentrations^14^, chemoattraction to Pi is likely related to bacterial virulence. Previous research has revealed several examples in which paralogous chemoreceptors of a given bacterial strain mediate taxis to the same ligand. Examples are the PctA and PctB chemoreceptors of *P. aeruginosa* for amino acids^22^,^23^ and McpS and McpQ of *P. putida* KT2440, which both respond to citrate^24^,^25^. For these examples, the receptors function via direct ligand recognition^23^,^24^,^25^.

**Figure 6:**
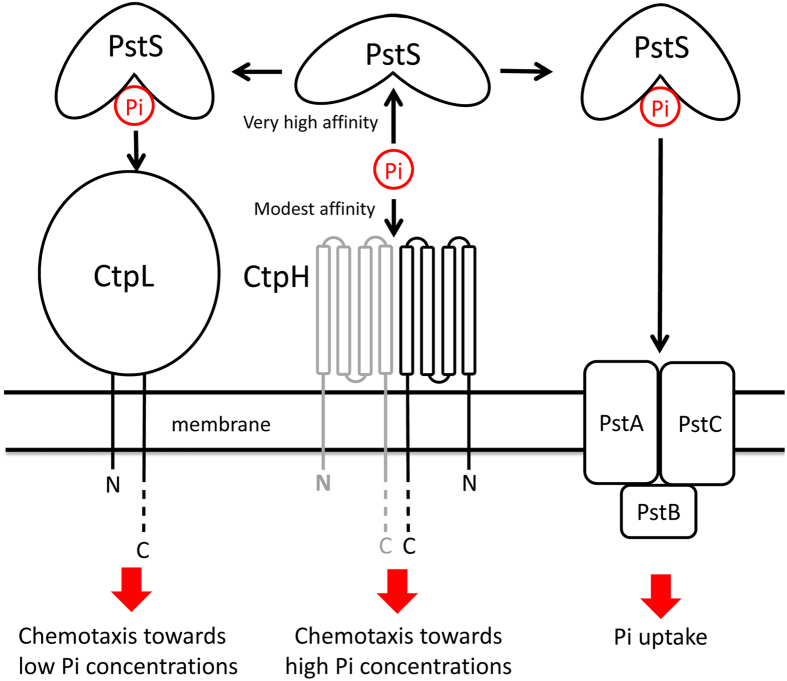
Model for the mechanism of Pi chemotaxis and transport in *P. aeruginosa*.

In marked contrast, Pi chemotaxis in *P. aeruginosa* is mediated by two non-paralogous receptors that differ in the type of LBD and, as we show here, in their mechanism. Our data have enabled the establishment of a model for Pi taxis and transport (Fig. 6).

Pi is recognized directly by CtpH and the modest affinity may account for the fact that this system responds to high Pi concentrations. CtpL does not recognize Pi directly, but via a complex between the periplasmic PstS and Pi. The ultra-tight binding of Pi to PstS may in turn explain why CtpL mediates taxis to very low concentrations of Pi.

We show here that CtpH-LBD binds Pi directly and that binding causes dimer stabilization. In this context clear parallels exist to Tar that, as CtpH, has a 4-helix bundle type of LBD. Milligan and Koshland^26^ have shown that aspartate binds with similar affinities to full-length Tar and Tar-LBD. Importantly, aspartate binding enhanced dimer formation of Tar-LBD. The three-dimensional structure of Tar-LBD may provide the molecular basis for this observation^27^. Aspartate bound to a site close to the Tar-LBD dimer interface in a way that amino acids from both monomers establish direct contacts with bound aspartate, which in turn stabilizes the dimeric form of the protein. Our data suggest that Pi binds in a similar manner to CtpH-LBD.

These new insights into Pi chemotaxis beg the question, “why did this complex, two-pronged system evolve?” Part of the answer can be found in the fact that each chemoreceptor is characterized by a dynamic range, which represents the ligand concentration at which the receptor mediates taxis^28^. The coexistence of two different chemoreceptors with different chemoattractant sensitivities can thus be considered a mechanism by which a microorganism can expand the dynamic range of chemotaxis towards a given chemoattractant. Other ways of broadening the dynamic range of a chemoreceptor have been reported, which consist in ligand recognition with negative cooperativity at the chemoreceptor dimer^29^ or in a covalent modification of the chemoreceptors which in turn alters ligand affinity^30^.

A second reason may be because of the dual function of PstS, which also feeds Pi into the Pi transporter. Pi has an enormous impact on *pstS* expression, as evidenced by the 223-fold increase in *pstS* expression that is observed when *P. aeruginosa* is grown in the presence of 0.2 mM versus 1 mM Pi^6^. This change in expression is of a much larger magnitude than those seen for the *pstC/A/B* transporter and *ctpH/L* genes, which increase in expression by 3 to 27-fold, respectively^6^. The regulation of *pstS* expression is thus a potent means for the coordinated regulation of both Pi chemotaxis and Pi transport. The concerted regulation of chemotaxis and transport may enable organisms to adapt to low Pi containing habitats.

In enterobacteria there are several cases where indirect chemoeffector recognition at chemoreceptors has been demonstrated in an unambiguous manner. Most of these examples are related to sugar chemotaxis. It was shown that the galactose/glucose- and the ribose-binding proteins of *E. coli*^31^,^32^,^33^ and *Salmonella typhimurium*^34^,^35^ were found to provide the chemoeffector to the Trg receptor^36^. In analogy, the maltose-binding protein^32^,^37^ was identified to stimulate the Tar receptor of *E. coli*^38^. In addition, indirect chemoeffector recognition in enterobacteria has also been observed for other compound classes such as the stimulation of the *E. coli* Tap and Tsr receptors by the dipeptide-binding protein DppA^39^ or the LsrB AI-2-binding protein^40^, respectively.

Interesting parallels exist between CtpL-PstS and the enterobacterial systems: 1) PstS and the enterobacterial periplasmic binding proteins involved in chemotaxis share significant structural similarities (Fig. S7); 2) the genes of the galactose/glucose-41, ribose^42^- and maltose^43^-binding proteins, DppA^44^ and LsrB^45^ as well as that of PstS^19^ are associated with the genes that encode the respective transporters and the resulting proteins provide the ligand to the membrane bound transporter. These periplasmic binding proteins serve therefore dual functions—they feed their cognate ligands to the transporter and the corresponding chemoreceptor^46^, and; 3) the expression levels of the sugar binding proteins and *pstS* are greatly affected by the concentration of cognate ligands^6^,^47^. Therefore, the transcriptional control of periplasmic ligand binding proteins may enable a concerted regulation of uptake and chemotaxis.

As stated above, periplasmic binding protein based receptor activation has been observed for the Trg, Tar, Tap and Tsr receptors^31^,^32^,^33^,^34^,^35^,^37^,^39^,^40^. These receptors possess a 4-helix bundle LBD of approximately 150–160 amino acids^27^. Our data show that periplasmic protein based receptor activation is not restricted to this receptor family, since CtpL-LBD does not form a 4-helix bundle fold. CtpL-LBD is with 286 amino acids significantly larger than a 4-helix bundle and was predicted to contain 9 helices (Fig. S2). Analysis of the CtpL-LBD sequence by the Phyre^2^ fold recognition server^48^ did not provide any information on the domain fold.

Periplasmic binding protein mediated chemotaxis mechanisms were unambiguously identified in the enterobacteria *E. coli* and *S. typhimurium*. The discovery of a similar mechanism in a bacterium of a different order, involving a different type of chemoreceptor and mediating taxis to a different chemoeffector suggests that such systems are widespread in nature. This work will lead the way for further investigations aimed at studying Pi chemotaxis within other bacterial pathogens.

## Materials and Methods

### Materials, bacterial strains and plasmids

Pi refers to K_2_HPO_4_ (Sigma). The strains and plasmids used are listed in Table S2.

### Cloning of *pstS, ctpH-LBD* and *ctpL-LBD*

The DNA fragments encoding PstS (PA5369) as well as amino acids 60–214 and 27–324 of CtpH (PA2561) and CtpL (PA4844), respectively, were amplified using the primers listed in Table S3. PCR products were digested with the respective enzymes and cloned into pET28b(+). For complementation purposes, the *pstS* gene was amplified from genomic DNA using primers pstS-pBBRMCS5-f and pstS-pBBRMCS5-r and cloned into pBBR1MCS-5 (Table S2).

### Protein expression and purification

*E. coli* BL21 (DE3) containing the expression plasmids were cultured in LB medium supplemented with 50 μg ml^−1^ kanamycin at 30 °C until an OD_660_ of 0.6, at which point 0.1 mM IPTG was added. Growth was continued at 18 °C overnight prior to cell harvest by centrifugation at 10,000 × g for 30 min. Pellets were resuspended in buffer A (30 mM Tris, 300 mM NaCl, 10 mM imidazole, 5% (v/v) glycerol, pH 7.0) and broken by French press at 1000 psi. After centrifugation at 20,000 × g for 1 hour, the supernatant was loaded onto a HisTrap column (Amersham Bioscience), washed with buffer A and proteins were eluted with a 45–1000 mM imidazole gradient in buffer A. To eliminate potentially bound compounds, PstS and CtpL-LBD were dialyzed against 10 mM Tris/HCl, 6 M guanidine hydrochloride, pH 8.0. For these experiments highest possible purity reagents were used (note: When the procedure was carried out with PstS and standard purity guanidine hydrochloride, no Pi binding was observed in ITC, which is due to the capture of Pi, present as an impurity in GdnHCl, during protein refolding). Protein was diluted to 10 μM and refolded by two consecutive dialyses into 10 mM Tris/HCl, pH 8.0. Aggregated protein corresponding to misfolded species was removed by filtration using 0.22 μm cut-off filters.

### CD spectroscopy

Experiments were performed using a Jasco J-715 (Tokyo) spectropolarimeter. Measurements were made at 25 °C with a 0.1 cm path-length quartz cuvette using a bandwidth of 1 nm, a scan rate of 100 nm.min^−1^ and a response time of 1 second. Data shown correspond to averages of 5 individual spectra.

### Intrinsic fluorescence spectroscopy

Measurements were made on a Cary Eclipse spectrofluorimeter (Varian) at 25 °C. An excitation wavelength of 280 nm was used, and emission spectra were recorded between 300 and 450 nm. Slit widths of 5 nm were used for both excitation and emission. Baselines obtained from samples containing only buffer were subtracted from all the data reported and the fluorescence intensities were normalized by the protein concentration.

### Dynamic Light Scattering

Measurements were made at 25 °C on a DynaPro MS-X instrument (Wyatt Technology Corporation, Santa Barbara, CA, USA) using a 30 μl quartz cuvette. Dynamics software (Wyatt Technology Corporation, Santa Barbara, CA, USA) was used for data collection and analysis. Data shown are means of 50 acquisitions. The change in viscosity for the denatured protein (due to the presence of 6 M GdnHCl) was taken into account in the data analysis.

### DSC

Experiments were carried out with a VP-DSC microcalorimeter (Malvern Instruments) at a rate of 60 °C/h. Proteins were in polybuffer (5 mM Tris, 5 mM Pipes, 5 mM Mes, pH 7.0). The molar partial heat capacity curves (Cp) were calculated from the DSC data and analyzed using Origin 8.5 (OriginLab, Northampton, MA) according to the two-state unfolding model as described in^49^. The errors have been estimated from the fittings as 95% confidence intervals for each parameter^50^.

### ITC

Experiments were conducted on a VP-microcalorimeter (Malvern Instruments) at 25 °C. CtpH-LBD and CtpL-LBD were dialyzed against polybuffer and PstS against 10 mM Tris/HCl, pH 8.0. Typically, 60–180 μM of CtpH-LBD and CtpL-LBD and 5–20 μM of PstS protein were titrated with 0.2–5 mM effector solutions. The mean enthalpies from the injection of ligands into buffer were subtracted from titration data prior to analysis with the “One binding site model” of ORIGIN following the algorithm described in^51^.

### AUC

Experiments were performed at 6 °C in a Beckman Coulter Optima XL-I analytical ultracentrifuge (Beckman-Coulter), using an An50Ti 8-hole rotor and 12 mm path-length charcoal-filled epon double-sector centrepieces. Samples were dialyzed into polybuffer in the presence or absence of Pi. Sedimentation velocity (SV) runs were carried out at 45,000 rpm using 400 μl samples. The *c*(*s*) method^52^ implemented in the SEDFIT v14.1 software was used. Buffer density (ρ = 1.016 g/mL) and viscosity (η = 0.0177 Poise) were determined by an Anton Paar Density Meter DMA 5000 M and Microviscometer Lovis 2000 ME. The partial specific volumes were calculated from the protein sequence using SEDNTERP software^53^. Sedimentation equilibrium (SE) data were acquired for 180 μl samples at speeds of 11,800, 18,100 and 31,000 rpm in the absorbance mode. The SE data were fitted using the SEDPHAT v10.55b software^54^. Errors shown are the errors of the fit, calculated as the standard deviations using a MonteCarlo analysis with a confidence level of 0.68. This procedure is implemented in SEDPHAT^54^.

### Pull-down experiments

*P. aeruginosa* PAO1 was grown in T_0_ medium^13^. Pellets were resuspended in buffer B (30 mM Tris/HCl, 300 mM NaCl, 10 mM imidazole, 5% (v/v) glycerol, 5 mM Pi, pH 7.0) and broken by French press. After centrifugation at 20,000 x g for 1 hour, the supernatant was loaded onto a HisTrap column on which CtpL-LBD had previously been immobilized. The column was washed with buffer B prior to protein elution using a 0–6 M guanidine hydrochloride gradient in buffer B. As a control, the *P. aeruginosa* PAO1 supernatant was applied to a column that did not contain CtpL-LBD. Bands of interest were excised from an SDS-PAGE gel, digested with trypsin and analyzed by MALDI-TOF mass spectrometry. The protein identity was established using the MASCOT software.

### Construction of *pstS* and *ctpH/pstS* mutants

In-frame deletion mutants defective in *pstS* were constructed by homologous recombination using a derivative plasmid of the suicide vector pKNG101. The resulting plasmid, pKNG101-5369UpDw, was generated by amplifying the up- and downstream flanking regions of the *pstS* and transferred to *P. aeruginosa* PAO1 strains by triparental conjugation using *E. coli* CC118λ*pir* and *E. coli* HH26 (pNJ500) as helper. Cointegrate selection was accomplished using M9 minimal medium supplemented with 10 mM succinate and 2 mg/ml streptomycin. To select derivatives that had undergone a second cross-over event during marker exchange mutagenesis, sucrose was added to a final concentration of 10% (w/v). Final mutants were confirmed by PCR and sequencing.

### Chemotaxis assays

T_0_ medium^14^ was inoculated with an overnight culture of *P. aeruginosa* PAO1 and incubated at 37 °C for 4 hours. The cells were washed twice with 10 mM HEPES, pH 7.0 and subsequently diluted into HEPES to an OD_600_ of 0.04. For filling, capillaries (Microcaps, Drummond Scientific) were heat-sealed at one end, warmed over the flame and the open end inserted into the chemoattractant solution. HEPES buffer containing capillaries were used as control. Bacterial suspensions were placed into the wells of Elisa plates, the capillaries inserted into the wells and incubated for 30 minutes. After capillary extraction, the open end was rinsed with water and placed into a microfuge tube containing 1 ml M9 medium supplemented with 15 mM succinate. The sealed end was broken and the contents emptied into the tube by a short centrifugation. Twenty microliters of the cell suspension was plated onto agar plates containing M9 medium and 15 mM succinate. Plates were incubated at 37 °C and colonies counted after 24 h.

## Additional Information

**How to cite this article**: Rico-Jiménez, M. *et al*. Two different mechanisms mediate chemotaxis to inorganic phosphate in *Pseudomonas aeruginosa.* *Sci. Rep.* **6**, 28967; doi: 10.1038/srep28967 (2016).

## Supplementary Material

Supplementary Information

## Figures and Tables

**Table 1:** Thermodynamic parameters for the titration of CtpH-LBD, CtpL-LBD and PstS with different ligands.

**Ligand 1**	**Ligand 2**	***K***_**D**_ **μM**	**Δ*****H*** **kcal/mol**
CtpH-LBD	Pi	22 ± 1	−5.7 ± 0.2
	pyrophosphate	31 ± 6	−0.9 ± 0.1
	ATP	Binding^1^	
	ADP	103 ± 21	−2.7 ± 1
	AMP	No binding	
	DL-glyceraldehyde 3-phosphate	No binding	
	D-fructose 6-phosphate	No binding	
	D-glucose 6-phosphate	No binding	
	phosphoenolpyruvate	No binding	
	6-phosphogluconate	No binding	
	arsenate	No binding	
	arsenite	No binding	
PstS^1^	Pi	0.007 ± 0.001	−5.1 ± 0.1
	pyrophosphate	2.6 ± 0.3	−8.3 ± 0.3
	ATP	0.3 ± 0.1	−2.9 ± 0.1
	CtpL-LBD	3.7 ± 0.3	−3.1 ± 0.1
CtpL-LBD	Pi	No binding	

Data are means and standard deviations from three experiments. ^1^A binding isotherm was observed but data analysis with different models failed.
