# Supplementary material for: Two different mechanisms mediate chemotaxis to inorganic phosphate in Pseudomonas aeruginosa
Source: Sci Rep. 2016 Jun 29;6:28967. doi: 10.1038/srep28967 (PMC4926252; doi:10.1038/srep28967)
Supplement: Supplementary Information [file srep28967-s1.pdf]

Supplementary Material

To

Two different mechanisms mediate chemotaxis to inorganic phosphate in *Pseudomonas aeruginosa*

Miriam Rico-Jiménez, Jose Antonio Reyes-Darias, Álvaro Ortega, Ana Isabel Díez Peña, Bertrand Morel and Tino Krell

CtpH

MPASPGHRDVLGCLVAACVPVQPGNPSRRSMLQQSLRAQILVLLGGSLAALLLIALACFGSLTGDVR
AYRELLGGPVRAAQLIDEANLQFRGQVQEWKNVLLRGRQTEAQTKYWSQFEAQERAVQDILGRLGSV
AEGELKDRVERLREEHRRLGTAYRQGRQRFLEAGADPIAGDQAVTGIDRATTAQMQTLRDELHQASD
LHSSSISAEARRTMLLGSLVLIGASLAVALLSLWLVNRNLVRPVQRLIEHIAQLSHGDFGERIEIRR
KDELGKLALAANTLRDFLVDIFDRLRRSTRDLDSASGSLNAIASLMAAGTREQFSRTDQVATAMQEM
SATAQEVARYAGDAARAADEADDSAQRGEDVMEETIRSIGEMRKEIDHTVEVIRQLESDSGRIGKVL
DVIRGIAEQTNLLALNAAIEAARAGDAGRGFAVVADEVRTLAQRTAESIAEIHQIIDTVQNGAVNAA
RAIESGQSRSEAGAEQVANAGAMLRQITASVESIRDMNRQIATAAEEQTAVAEEISRNLTEIASIAS
SNQEQVEQTEAASRDLHGLSAQLGDALQRLRA

CtpL

MRLKQLTNLNTLLLLTVCLALGITLWWSQRAMERPFQLLDQYLELSQRFDEQVARNIRQYLGSGDAV
RQQAALQALESLAEALPELPPDLARTLAPSLAELREFSAGDLLAAGKLAGDPQGLLLQAERDLTGNL
EQWSAYLDAAAGQPQAGAYRTPLLLASLHLTRLSLARAKLVESANPALAGDVERELANLREQAGRIE
ALPLLGVLDEQRSASDDFAAMMGLAGDAEAGAGNAEDRGVALRRELASLLQRYPDELRRTRDLIERR
QQLSADTGARLDAVRQALATLEPQVRGERQRLQGQVRLIQGGMIALILLIALAIDSLQRRLARVLGQ
LVPALSAWADGDFSRPISLRTRTEDLRNLEDSLNRLRSFLAELVGAIHRRAEQVAGSSQTLAEVSSG
LHAGVERQAGDTGQIRDALGDMEAAIQQVAGDASQTADASRSAGQAVEHGQRVIGESLGGLRELVDE
VQGNAQSIERLAEESATIGSVLTVIRSIAEQTNLLALNAAIEAARAGDQGRGFAVVAEEVRSLAQRT
AGATEEIQQLIGRLQQAARQSVEAMRSQVEHAERTAEQAGAAEGALDEVVAAIHTIGVMAERIAEGS
TQQSQAVGEIRSHSERIHALGGENLRLIGHSREQGEQLRQLGGDLRTTVQAFRL

Fig. S1) Prediction of CtpH and CtpL transmembrane regions (in red) via the dense alignment surface method [1]. The ligand binding domains of CtpH and CtpL (155 and 286 amino acids, respectively) are shaded in yellow.

CtpH-LBD

```
                 10        20        30        40        50        60        70
                  |         |         |         |         |         |         |
UNK_14480 SLTGDVRAYRELLGGPVRAAQLIDEANLQFRGQVQEWKNVLLRGRQTEAQTKYWSQFEAQERAVQDILGR
DSC       cccchhhhhhhhhhhhhhhhhhhhhhhhhhhhhhhhhhhhhhhcccchhhhhhhhhhhhhhhhhhhhhhh
MLRC      cccchhhhhhhhhccchhhhhhhhhhhhhhhhhhhhhhhhhhcccccchhhhhhhhhhhhhhhhhhhhhh
PHD       cccchhhhhhhhhhccchhhhhhhhhhhhhhhhhhhhhhhhhccccchhhhhhhhhhhhhhhhhhhhhhh
Sec.Cons. cccchhhhhhhhhhcchhhhhhhhhhhhhhhhhhhhhhhhhhccccchhhhhhhhhhhhhhhhhhhhhhh

                 80        90       100       110       120       130       140
                  |         |         |         |         |         |         |
UNK_14480 LGSVAEGELKDRVERLREEHRRLGTAYRQGRQRFLEAGADPIAGDQAVTGIDRATTAQMQTLRDELHQAS
DSC       ccccccchhhhhhhhhhhhhhhhhhhhhhhhhhhhhhhccccchhhhhhhhhhhhhhhhhhhhhhhhhhhh
MLRC      hhccchhhhhhhhhhhhhhhhhhhhhhhhhhhhhhhcccccccccccccccccchhhhhhhhhhhhhhhh
PHD       hhcccccchhhhhhhhhhhhhhhhhhhhhhhhhhhhhcccccccccccccccccchhhhhhhhhhhhhhh
Sec.Cons. hhccccchhhhhhhhhhhhhhhhhhhhhhhhhhhhhhccccccccccccccccchhhhhhhhhhhhhhhh

                150
                  |
UNK_14480 DLHSSSISAEARRTM
DSC       hhhhhhhhhhhhccc
MLRC      hhhhhhhhhhhhhcc
PHD       hhhhhhhhhhhhccc
Sec.Cons. hhhhhhhhhhhhccc
```

CtpL-LBD

```
                 10        20        30        40        50        60        70
                  |         |         |         |         |         |         |
UNK_28340 TLWWSQRAMERPFQLLDQYLELSQRFDEQVARNIRQYLGSGDAVRQQAALQALESLAEALPELPPDLART
DSC       ccchhhhhhhhhhhhhhhhhhhhhhhhhhhhhhhhhhcccccchhhhhhhhhhhhhhhhhcccccchhhhh
MLRC      cccchhhhhhhhhhhhhhhhhhhhhhhhhhhhhhhhhhcccchhhhhhhhhhhhhhhhhhhhcchhhhhh
PHD       cchhhhhhhhhhhhhhhhhhhhhhhhhhhhhhhhhhhhccchhhhhhhhhhhhhhhhhhhhccchhhhhh
Sec.Cons. ccchhhhhhhhhhhhhhhhhhhhhhhhhhhhhhhhhhhcccchhhhhhhhhhhhhhhhhhhccchhhhhh

                 80        90       100       110       120       130       140
                  |         |         |         |         |         |         |
UNK_28340 LAPSLAELREFSAGDLLAAGKLAGDPQGLLLQAERDLTGNLEQWSAYLDAAAGQPQAGAYRTPLLLASLH
DSC       hhhhhhhhhhhhhhhhhhhhhccccchhhhhhhhhhhhhhhhhhhhhcccccccchhhhhhhhhhhhhhh
MLRC      hhhhhhhhhhhccchhhhhhhcccccchhhhhhhhhhhhhhhhhhhhhhhccccccchhhhhhhhhhhhh
PHD       hhhhhhhhhhcccchhhhhcccccchhhhhhhhhhhhhhhhhhhhhhhhhhccccchhhhhhhhhhhhhh
Sec.Cons. hhhhhhhhhhhccchhhhhhhccccchhhhhhhhhhhhhhhhhhhhhhhhcccccchhhhhhhhhhhhhh

                150       160       170       180       190       200       210
                  |         |         |         |         |         |         |
UNK_28340 LTRLSLARAKLVESANPALAGDVERELANLREQAGRIEALPLLGVLDEQRSASDDFAAMMGLAGDAEAGA
DSC       hhhhhhhhhhhhhhcchhhhhhhhhhhhhhhhhhhhhhhhhhcccccccccchhhhhhhhhhccccccccc
MLRC      hhhhhhhhhhhhhcccchhhhhhhhhhhhhhhhhhhhhhccccceeccccccccchhhhhhhhccccccccc
PHD       hhhhhhhhhhhhhhhchhhhhhhhhhhhhhhhhhhhhhhcccccceccccchhhhhhhhccccccccccc
Sec.Cons. hhhhhhhhhhhhhhcchhhhhhhhhhhhhhhhhhhhhhhcccccccccccchhhhhhhhhhccccccccc

                220       230       240       250       260       270       280
                  |         |         |         |         |         |         |
UNK_28340 GNAEDRGVALRRELASLLQRYPDELRRTRDLIERRQQLSADTGARLDAVRQALATLEPQVRGERQRLQGQ
DSC       ccchhhhhhhhhhhhhhhhhhhhhhhhhhhhhhhhhhhhhhhhhhhhhhhhhhhhhhhhhhhhhhhhhh
MLRC      cccchhhhhhhhhhhhhhhhchhhhhhhhhhhhhhhhhhhhhhhhhhhhhhhhhhhhhhhhhhhhhhhh
PHD       cchhhhchhhhhhhhhhhhhhhhhhhhhhhhhhhhhhhhhhhhhhhhhhhhhhhhhhhhhhhhhhhhhh
Sec.Cons. ccchhhhhhhhhhhhhhhhhhhhhhhhhhhhhhhhhhhhhhhhhhhhhhhhhhhhhhhhhhhhhhhhhh

UNK_28340 VRLIQG
DSC       hhhccc
MLRC      hhhhcc
PHD       hhhhcc
Sec.Cons. hhhhcc
```

Fig. S2) Secondary structure prediction of the ligand binding domains of CtpH and CtpL using the consensus method from the Network Protein Sequence Analysis (NPSA) [2]. h= α-helix, c=coil, e=β-strand.

```
                   10         20         30         40         50         60
                    |          |          |          |          |          |
CtpL-LBD     TLWWSQRAMERPFQLLDQYLELSQRFDEQVARNIRQYLGSGDAVRQQAALQALESLAEAL
CtpH-LBD     ------------------------------------------------------------

                   70         80         90        100        110        120
                    |          |          |          |          |          |
CtpL-LBD     PELPPDLARTLAPSLAELREFSAGDLLAAGKLAGDPQGLLLQAERDLTGNLEQWSAYLDA
CtpH-LBD     ---------SLTGDVRAYRELLGGPVRAA--------QLIDEANLQFRGQVQEWKNVLLR

                  130        140        150        160        170        180
                    |          |          |          |          |          |
CtpL-LBD     AAGQPQAGAYRTPLLLASLHLTRLSLARAKLVESANPALAGDVERELANLREQAGRIEAL
CtpH-LBD     GRQTEAQTKYWS-------QFEAQERAVQDILGRLGSVAEGELKDRVERLREEHRRLGTA

                  190        200        210        220        230        240
                    |          |          |          |          |          |
CtpL-LBD     PLLGVLDEQRSASDDFAAMMGLAGDAEAGAGNAEDRGVALRRELASLLQRYPDELRRTRD
CtpH-LBD     YRQGRQRFLEAGADPIAGDQAVTG---------------IDRATTAQMQTLRDELHQASD

                  250        260        270        280
                    |          |          |          |
CtpL-LBD     LIERRQQLSADTGARLDAVRQALATLEPQVRGERQRLQGQVRLIQG
CtpH-LBD     LHS--SSISAEARRTM------------------------------
```

Fig. S3) Sequence alignment of the ligand binding domains of the CtpH and CtpL receptors. Alignment was carried out using default parameters in CLUSTALW from the Network Protein Sequence Analysis suite (http://npsa-pbil.ibcp.fr/cgi-bin/npsa_automat.pl?page=/NPSA/npsa_clustalw.html).

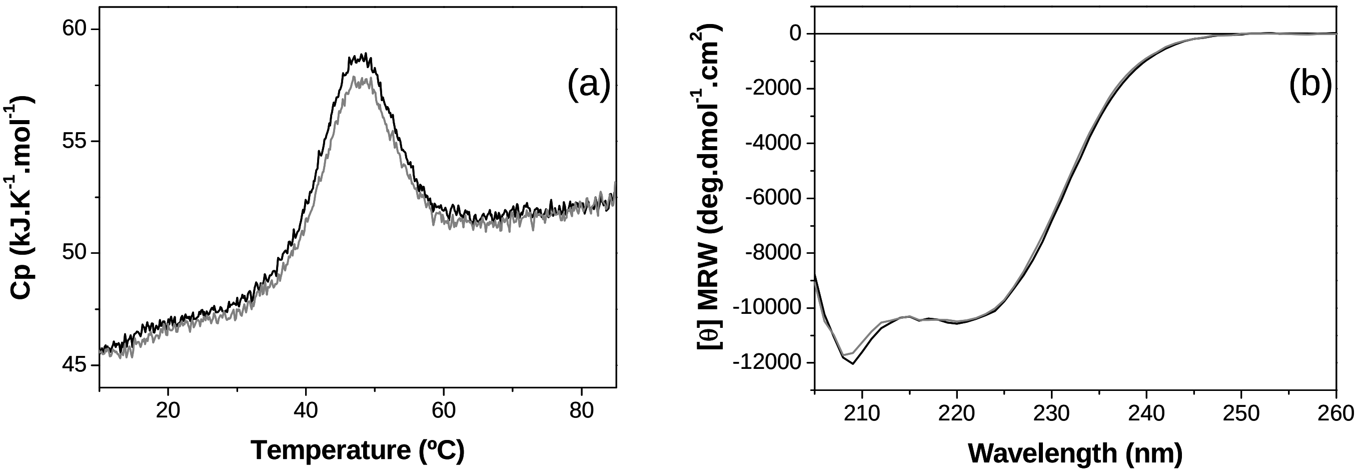

Fig. S4) Reversibility of the CtpH-LBD unfolding process. (a) DSC thermogram of CtpH-LBD (black line) and second consecutive up-scan of the same sample (grey line). (b) Far UV CD spectra of CtpH-LBD before (black line) and after heat treatment at 85ºC for 5 minutes (grey line).

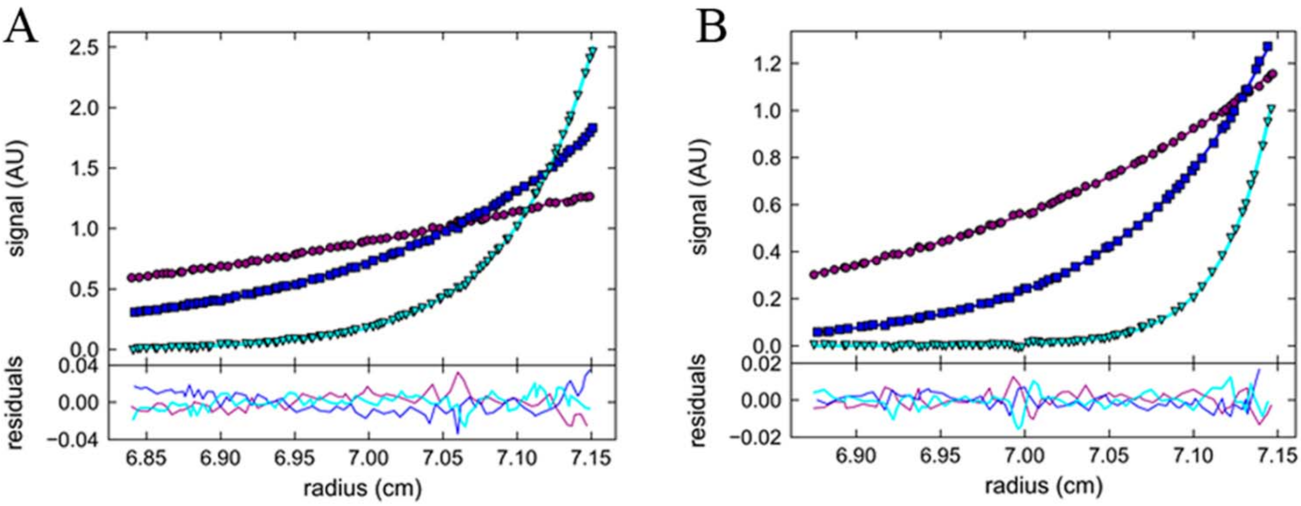

Fig. S5) Sedimentation equilibrium analytical ultracentrifugation analysis of CtpH-LBD. Gradients recorded using absorbance at 280 nm for 45 µM CtpH-LBD in the absence (A) or in the presence (B) of 0.5 mM Pi. Measurements were made at 6 °C and at velocities of 11800 rpm (cyan), 18100 rpm (blue) and 31000 rpm (purple). The lines represent best fits obtained with the SEDPHAT single species of interacting system model. The residuals for the fits are shown in the lower panel.

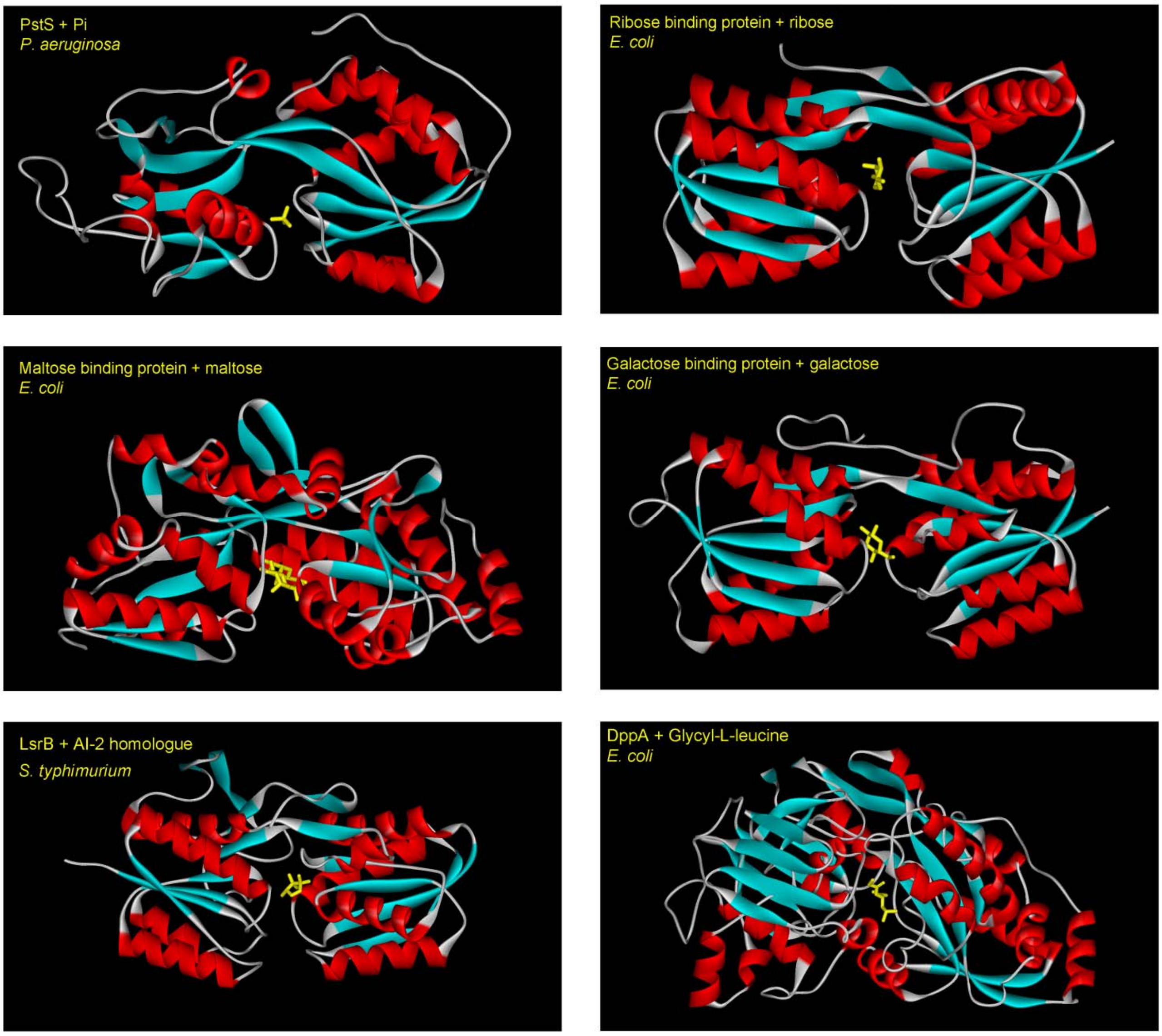

Fig. S6) Structures of periplasmic ligand binding proteins in complex with their cognate ligands (in yellow). PstS of *P. aeruginosa* in complex with Pi [3](pdb ID: 4OMB), Ribose Binding Protein (pdb ID: 2GX6), Galactose Binding Protein (pdb ID: 2GBP) [4], Maltose Binding Protein (pdb ID: 2MV0) [5], Dipeptide binding protein DppA (pdb ID: 1DPP) [6] and the LsrB protein in complex with an AI-2 homologue (pdb ID: 1TJY) [7].

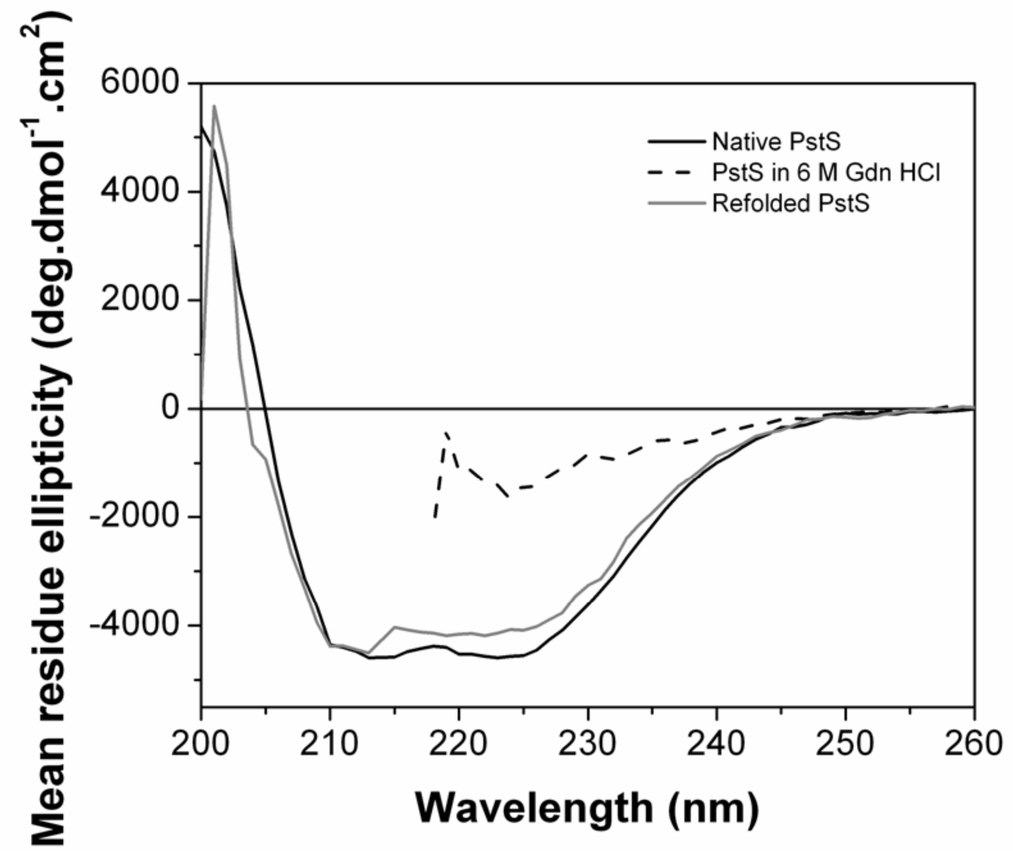

Fig. S7) Far UV circular dichroism spectra of PstS obtained after affinity purification (native PstS), unfolded protein (PstS in 6M GdnHCl) and after refolding (refolded PstS). Proteins were at a concentration of 10 µM in 10 mM Tris/HCl, pH 8.0 (native and refolded PstS) or in 10 mM Tris/HCl, pH 8.0, containing 6 M guanidine hydrochloride. The derived percentages of secondary structure elements are shown in Table S1.

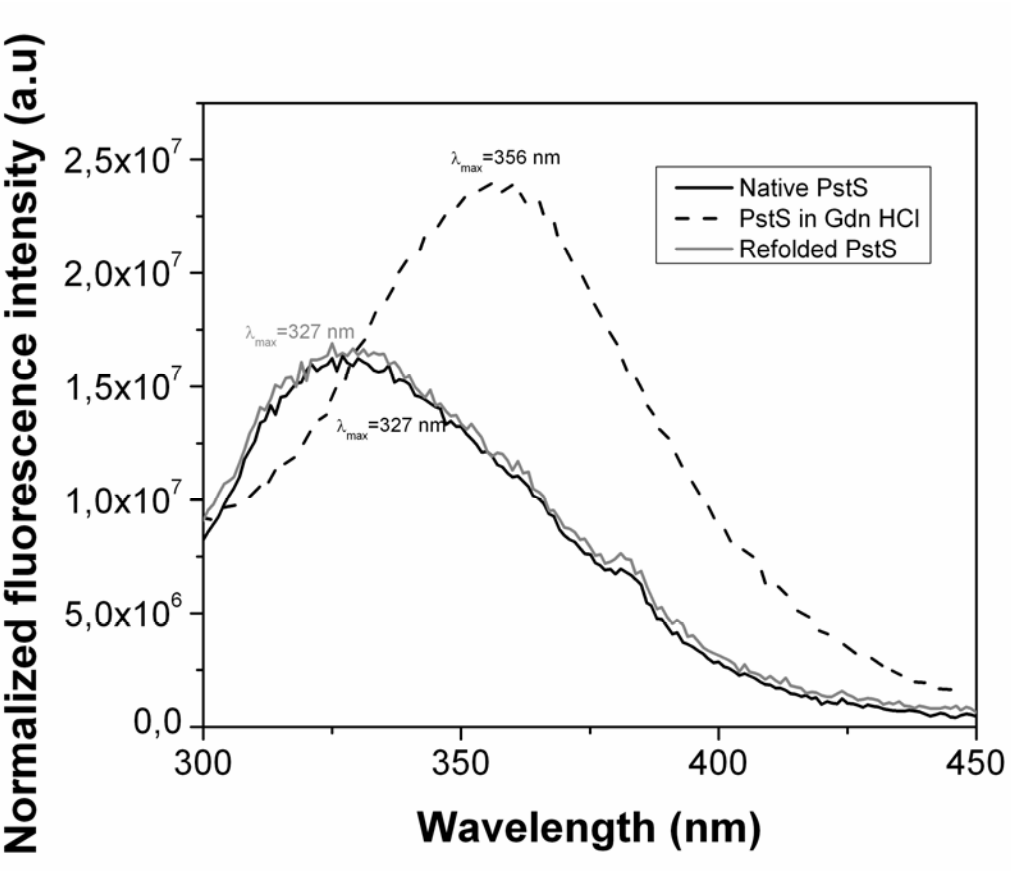

Fig. S8) Intrinsic fluorescence emission spectra of native PstS, unfolded protein (in 6 M guanidine hydrochloride) and after refolding. The wavelengths of the maximum fluorescence intensity are indicated. Proteins were at a concentration of 10 µM in 10 mM Tris/HCl, pH 8.0 (native and refolded PstS) or in 10 mM Tris/HCl, pH 8.0, containing 6 M guanidine hydrochloride.

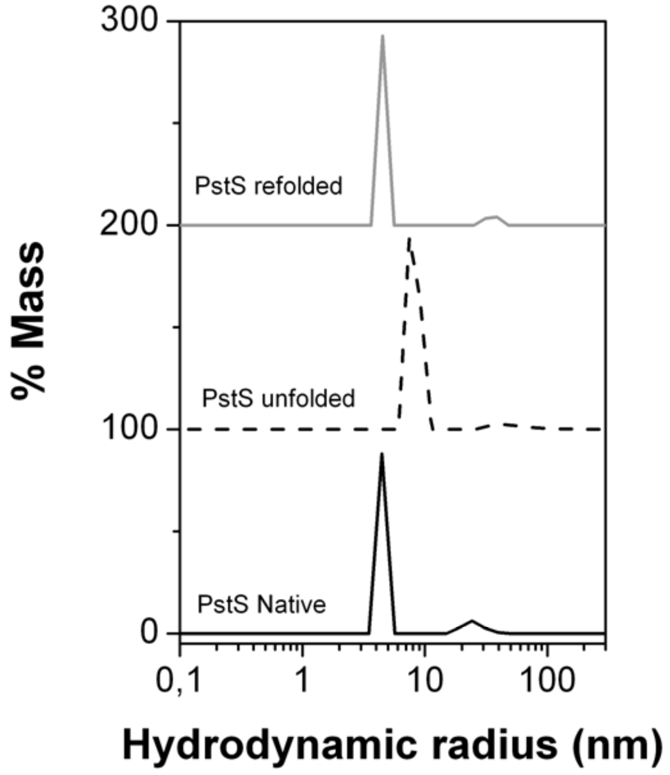

Fig. S9) Dynamic light scattering measurements of PstS in its native, unfolded (in guanidine hydrochloride) and refolded forms. For clarity traces have been moved arbitrarily on the y-axis. The following hydrodynamic radii were determined: *refolded PstS:* Rh=4.5 nm (92.7 %) and 35.3 nm (7.3 %); *unfolded PstS:* Rh=8.2 nm (94.1 %) and 54.6 nm (5.9 %); *native PstS:* Rh=4.4 nm (88.1 %) and 25.1 nm (11.9 %).

Table S1 : Secondary structure contents of native, unfolded and refolded PstS as determined by the deconvolution of the far UV CD dichroism spectra shown in Fig. S7. Spectra were deconvoluted using the CDNN program [8] and the errors were obtained using the deconvolution results obtained in the 260-205 nm range and those in the 260-210 nm range.

	Native PstS (%)	Unfolded PstS*	Refolded PstS (%)
α -helix	19.1 ± 1.4	n.a.	18.4 ± 1.0
β -antiparallel	10.3 ± 0.6	n.a.	10.9 ± 0.9
β -parallel	10.8 ± 0.7	n.a.	11.0 ± 0.7
β -turn	12.7 ± 0.1	n.a.	12.4 ± 0.1
random coil	47.1 ± 1.0	n.a.	47.3± 1.0

*Not analysed. Data could not be deconvoluted due to reduced wavelength range.

Table S2) Strains and Plasmids used in this study.

Bacterial strains	Relevant characteristics[a]	Reference
Escherichia coli		
DH5α	*supE44 lacU169* (*Ø80lacZΔ M15*) *hsdR17* ($r_k^- m_k^-$) *recA1 endA1 gyrA96 thi-1 relA1*	[9]
BL21 (DE3)	F^- *ompI hsdS*$_B$ (r_B^- m_B^-)	[10]
HB101	F^- *mcrB mrr hsdS20*(r_B^- m_B^-) *recA13 leuB6 ara-14 proA2 lacY1 galK2 xyl-5 mtl-1 rpsL20*(Sm^R) *glnV44* λ^-	[11,12]
CC118λ*pir*	Rif^R; Δ(*ara-leu*) *araD* Δ*lacX74 galE galK phoA20 thi-1 rpsE rpoB argE*(Am) *recA1* Tn*7* λpir	[13]
Pseudomonas aeruginosa		
PAO1	Wild type	[14]
ctpH	PAO1, *ctpH*::Km; Km^R	[15]
pstS	PAO1 in-frame Δ*pstS*	This study
ctpHpstS	PAO1 in-frame Δ*pstS*, *ctpH*::Km; Km^R	This study
**Plasmids**		
pET28b(+)	Km^R; protein expression vector	Novagen
pET28b-CtpH-LBD	Km^R; pET28b(+) derivative encoding CtpH-LBD (amino acids 60-214)	This work
pET28b-CtpL-LBD	Km^R; pET28b(+) derivative encoding CtpL-LBD (amino acids 27-324)	This work
pET28b-PstS-LBD	Km^R; pET28b(+) derivative with *pstS* gene	This work
pRK600	Cm^R; *mob tra*	[12]
pUC18NotI	Ap^R; *ori* pMB1, pUC18 derivative but with NotI sites flanking the MCS; cloning vector	Biomedal
pUC18NotI-Up	Ap^R; pUC18NotI derivative containing a 0.35-kb HindIII-XbaI fragment of the upstream region of *pstS*	This study
pUC18NotI-Dw	Ap^R; pUC18NotI derivative containing a 0.35-kb EcoRI-XbaI fragment of the downstream region of *pstS*	
pUC18NotI-5369UpDw	Ap^R; a 0.7-kb HindIII-EcoRI fragment containing fused up- and downstream regions of *pstS* cloned in pUC18NotI	This study
pKNG101	Sm^R; *ori*R6K *mob sacBR*; suicide vector	[16]
pKNG101-UpDw	Sm^R; pKNG101derivative containing a 0.7-Kb NotI fragment from pUC18NotI-5369UpDw cloned into pKNG101	This study
pRK600	Cm^R; *ori*ColE1 RK2 *mob*$^+$ *tra*$^+$; helper plasmid	[17]
pBBR1MCS-5	Gm^R; *ori*RK2 *mob*$^+$	[18]
pBBR1MCS-5-pstS	Gm^R; pBBR1MCS-5 derivative containing *pstS*	This study

[a]Cm, chloramphenicol; Ap, ampicillin; Sm, streptomycin; Km, kanamycin; Gm, gentamycin; Rif, rifampicin

Table S3) Oligonucleotides used in this study. Restriction sites are underlined.

Name	Sequence	Purpose
CtpH-LBD-f	5´-AA<u>CATATG</u>ATGTCGCTGACCGGCGACGTACGCG-3´	Construction of pET28b-CtpH-LBD
CtpH-LBD-r	5´-AA<u>GGATCC</u>TCAGGTGCGCCGGGCCTCCGCGCTG-3´	Construction of pET28b-CtpH-LBD
CtpL-LBD-f	5´-AA<u>CATATG</u> ATGTCGCAACGCGCCATGGAGCGGC-3´	Construction of pET28b-CtpL-LBD
CtpL-LBD-r	5´-AA<u>GGATCC</u>TCACTGGATCAGGCGTACCTGGCCT-3´	Construction of pET28b-CtpL-LBD
PstS-f	5´-AA<u>CATATG</u>AAACTCAAGCGTTTGATGG-3´	Construction of pET28b-PstS-LBD
PstS-r	5´-AA<u>GAATTC</u>TTACAGGCCCAGTTCCTTG-3´	Construction of pET28b-PstS-LBD
PA5369 Upf	5´-AA<u>AAGCTT</u>GTCAGCGATGACCGGG-3´	Construction of pUC18NotI-5369Up
PA5369 Upr	5´-AA<u>TCTAGA</u>GCCAGTATCGTCAGGCCG-3´	Construction of pUC18NotI-5369Up
PA5369 Dwf	5´-AA<u>TCTAGA</u>TCCAGTGACTTGAGCGGAT-3´	Construction of pUC18NotI-5369Dw
PA5369 Dwr	5´-AA<u>GAATTC</u>TAGGCCAGGTAGAAGAAGATCA-3´	Construction of pUC18NotI-5369Dw
pstS-pBBRMCS5-f	5’-AGA<u>ATCGCA</u>TATGAAACTCAAGCGTTTGATGGC-3’	Construction of pBBR1MCS-5-pstS
pstS-pBBRMCS5-r	5’-AA<u>GAATTC</u>TTACAGGCCCAGTTCCTTGATCGC-3’	Construction of pBBR1MCS-5-pstS

References

1 Cserzo, M., Wallin, E., Simon, I., von Heijne, G. & Elofsson, A. Prediction of transmembrane alpha-helices in prokaryotic membrane proteins: the dense alignment surface method. *Protein Eng.* **10**, 673-676 (1997).

2 Deleage, G., Blanchet, C. & Geourjon, C. Protein structure prediction. Implications for the biologist. *Biochimie* **79**, 681-686 (1997).

3 Neznansky, A., Blus-Kadosh, I., Yerushalmi, G., Banin, E. & Opatowsky, Y. The Pseudomonas aeruginosa phosphate transport protein PstS plays a phosphate-independent role in biofilm formation. *FASEB J.* **28**, 5223-5233 (2014).

4 Vyas, N. K., Vyas, M. N. & Quiocho, F. A. Sugar and signal-transducer binding sites of the *Escherichia coli* galactose chemoreceptor protein. *Science* **242**, 1290-1295 (1988).

5 Shilton, B. H., Shuman, H. A. & Mowbray, S. L. Crystal structures and solution conformations of a dominant-negative mutant of *Escherichia coli* maltose-binding protein. *J. Mol. Biol.* **264**, 364-376 (1996).

6 Dunten, P. & Mowbray, S. L. Crystal structure of the dipeptide binding protein from *Escherichia coli* involved in active transport and chemotaxis. *Protein Sci.* **4**, 2327-2334 (1995).

7 Miller, S. T. *et al.* Salmonella typhimurium recognizes a chemically distinct form of the bacterial quorum-sensing signal AI-2. *Mol. Cell* **15**, 677-687 (2004).

8 Bohm, G., Muhr, R. & Jaenicke, R. Quantitative analysis of protein far UV circular dichroism spectra by neural networks. *Protein Eng.* **5**, 191-195 (1992).

9 Woodcock, D. M. *et al.* Quantitative evaluation of *Escherichia coli* host strains for tolerance to cytosine methylation in plasmid and phage recombinants. *Nucleic Acids Res.* **17**, 3469-3478 (1989).

10 Studier, F. W. & Moffatt, B. A. Use of bacteriophage T7 RNA polymerase to direct selective high-level expression of cloned genes. *J. Mol. Biol.* **189**, 113-130 (1986).

11 Boyer, H. W. & Roulland-Dussoix, D. A complementation analysis of the restriction and modification of DNA in *Escherichia coli*. *J. Mol. Biol.* **41**, 459-472 (1969).

12 Kessler, B., de Lorenzo, V. & Timmis, K. N. A general system to integrate *lacZ* fusions into the chromosomes of gram-negative eubacteria: regulation of the P_m promoter of the TOL plasmid studied with all controlling elements in monocopy. *Mol. Gen. Genet.* **233**, 293-301 (1992).

13 Herrero, M., de Lorenzo, V. & Timmis, K. N. Transposon vectors containing non-antibiotic resistance selection markers for cloning and stable chromosomal insertion of foreign genes in gram-negative bacteria. *J. Bacteriol.* **172**, 6557-6567 (1990).

14 Stover, C. K. *et al.* Complete genome sequence of *Pseudomonas aeruginosa* PAO1, an opportunistic pathogen. *Nature* **406**, 959-964 (2000).

15 Wu, H. *et al.* Identification and characterization of two chemotactic transducers for inorganic phosphate in *Pseudomonas aeruginosa*. *J Bacteriol* **182**, 3400-3404 (2000).

16 Kaniga, K., Delor, I. & Cornelis, G. R. A wide-host-range suicide vector for improving reverse genetics in gram-negative bacteria: inactivation of the *blaA* gene of *Yersinia enterocolitica*. *Gene* **109**, 137-141 (1991).

17 de Lorenzo, V., Herrero, M., Jakubzik, U. & Timmis, K. N. Mini-Tn5 transposon derivatives for insertion mutagenesis, promoter probing, and chromosomal insertion of cloned DNA in gram-negative eubacteria. *J. Bacteriol.* **172**, 6568-6572 (1990).

18 Kovach, M. E. *et al.* Four new derivatives of the broad-host-range cloning vector pBBR1MCS, carrying different antibiotic-resistance cassettes. *Gene* **166**, 175-176 (1995).
